# Prevalence and incrimination of *Anopheles fluviatilis* species S (Diptera: Culicidae) in a malaria endemic forest area of Chhattisgarh state, central India

**DOI:** 10.1186/1756-3305-5-215

**Published:** 2012-09-28

**Authors:** Nutan Nanda, Rajendra M Bhatt, Shri N Sharma, Pallab K Rana, Narayani P Kar, Akash Sharma, Tridibes Adak

**Affiliations:** 1 National Institute of Malaria Research, Sector 8, Dwarka, Delhi-110077, India; 2 National institute of Malaria Research, Field Unit, RLTRI Campus, Lalpur, Raipur, 492015, Chhattisgarh, India

**Keywords:** *Anopheles fluviatilis*, *Anopheles culicifacies*, Species complex, Sibling species, Anthropophagic, Zoophagic, Malaria vector

## Abstract

**Background:**

Chhattisgarh state in central India is highly endemic for malaria and contributes about 13% of annually reported malaria cases in the country with predominance of *P. falciparum*. Entomological investigations were carried out in a tribal forested area of district Bastar located in the southern part of Chhattisgarh state to record the prevalence of sibling species of *Anopheles fluviatilis* and *An. culicifacies* complexes. The vector species complexes were investigated at sibling species level for their biology in terms of resting and feeding behavior and malaria transmission potential.

**Methods:**

Indoor resting vector mosquitoes collected during 2010–2011 were identified to sibling species by cytotaxonomy and polymerase chain reaction (PCR) assay. The blood meal source analysis and incrimination studies were done at sibling species level by counter current immunoelectrophoresis and enzyme linked immunosorbent assay (ELISA) respectively.

**Results:**

Analysis of sibling species composition revealed predominance of *An. fluviatilis* species S in the study area, which was found to be highly anthropophagic and rested in human dwellings whereas the sympatric species T was primarily zoophagic. Incrimination studies showed high sporozoite rate in species S, thereby confirming its vectorial efficiency. *An. culicifacies* was encountered in low numbers and comprised species B and C in almost equal proportion. Both these species were found to be exclusively zoophagic.

**Conclusion:**

The observations made strongly suggest that species S of Fluviatilis Complex is the principal vector of malaria in certain forest areas of district Bastar, Chhattisgarh state and should be the target species for vector control operation. Vector control strategies based on biological characteristics of Fluviatilis S will lead to substantial decline in malaria incidence in such areas.

## Background

Chhattisgarh, the 26^th^ state of India was created from the eastern part of Madhya Pradesh in November 2000 with 16 revenue districts. The state having a population of 25.5 million (~2.11% of the country’s population) contributes about 13% of annually reported malaria cases in India [1] and the bulk of malaria burden is borne by tribal forested areas in the north and south. District Bastar situated in the southern part of Chhattisgarh state is highly endemic for malaria with annual parasite incidence (API-cases/1000 population/year) ranging from 16.6 to 28.2 during the last five years and *Plasmodium falciparum* accounting ~ 90% of total malaria cases (Directorate of Health Services, Govt. of Chhattisgarh). The malaria vector control activities implemented by The District Health Department include two rounds of indoor residual spraying (IRS) with synthetic pyrethroids, this is carried out between mid-June to mid-October in villages with API >2. The IRS operations are prioritized on the basis of the quantity of insecticides available/allotted to the district health authorities.

Tropical climate, thick forest cover, good rainfall, perennial rivers and streams provide ideal conditions for malaria transmission [2,3]. The area is inhabited by socio-economically backward tribal people. Out of 17 anopheline species reported from Bastar district [4]*Anopheles culicifacies* Giles, 1901 and *An. fluviatilis* James, 1902 are the important vectors of malaria in this region [3,5].

Both the above mentioned malaria vectors have been established as species complexes comprising cryptic/sibling species [6,7]. *Anopheles culicifacies* is a complex of 5 sibling species provisionally designated as A, B, C, D and E which vary in their biological characteristics and malaria transmission potential [8-11]. Similarly the Fluviatilis Complex comprises 4 sibling species S, T, U, and a new species V (Nanda et.al. unpublished observations) which exhibit distinct variations in their biology and vectorial potential [12-16]. Therefore, it is most important that the vector species complexes are investigated at sibling species level for their distribution pattern, relative proportion, biological characteristics and vectorial efficiency. This is essential in targeting particular species for vector control operation in an area and adopting an appropriate strategy.

In the present study surveys were undertaken in forested areas of district Bastar of Chhattisgarh state to identify the vector species prevalence and their biology in terms of resting behavior, feeding preference and vectorial potential. Results of the entomological investigations are presented in the paper.

## Methods

### Study Area

**Figure 1:**
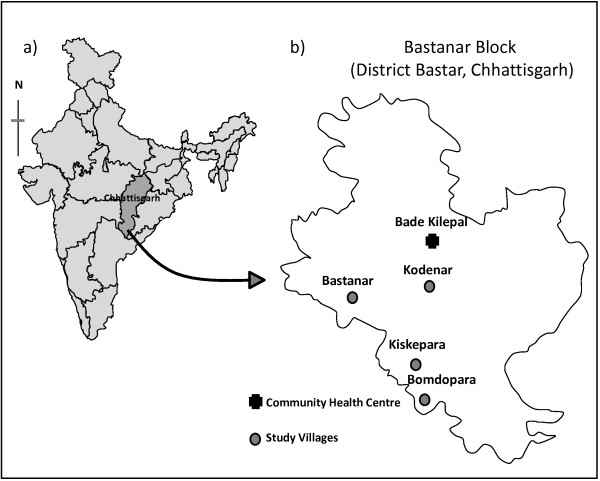
a) Map of India showing Chhattisgarh state, b) Bastanar block of district Bastar showing study villages under Bade Kilepal Primary Health Centre.

District Bastar lies in the southern part of Chhattisgarh state and extends from 19°15'N latitude to 81°40'E longitude (Figure 1). It has an area of 17016 sq km. and a population of 1422894 with predominance of tribals (67%). About 53.7% of the district area is covered with deciduous forest with largely tropical vegetation. The study villages included Bastanar, Kodenar, Kiskepara and Bomdopara under Bade Kilepal Primary Health Centre (PHC). The villages are situated in a forested hilly and foot hill area. The major breeding sites around these villages are slow running streams, their channels and water logged terraced paddy fields during monsoon and post-monsoon months. Breeding of *An. fluviatilis* has been reported from such habitats in the past [17]. The villages are inhabited by socio-economically backward tribal people depending upon subsistence agriculture and bartering of forest produce. The houses are mainly made of mud bricks with thatched roof, often with adjacent cattle sheds (open sheds with thatched roof supported by wooden poles and without walls). Cattle are generally tethered outside the houses in the open except during the rains. 

### Mosquito collection and processing

Indoor resting mosquitoes were collected from 10–12 human dwellings per village between 6.00 to 8.00 AM using a suction tube and torch light [18]. Mosquitoes were collected by employing two trained insect collectors for 4–5 days every month during January-February, November-December 2010 and November-December 2011, which is the peak prevalence period of *An. fluviatilis*. Collections were made from human dwellings only as cattle sheds were of open type and yielded no mosquitoes. The vector species *An. fluviatilis* and *An. culicifacies* were identified morphologically following standard keys [19,20] and their man-hour densities were calculated using the formula: number of mosquitoes collected x 60 / total collection time in minutes. Blood fed vectors were transferred into cloth cages and kept under ambient conditions. Cotton swabs soaked with 10% glucose solution were provided to allow vectors to reach half-gravid stage. Ovaries were removed from individual half gravid vectors (in Christophers stage late III) and preserved in modified Carnoy’s fixative (1:3 acetic acid: methanol). Blood from the mid-gut was smeared on Whatman No.1 filter paper (Whatman International Inc., Maidstone; England) for identification of blood meal source. Head and thorax were separated and stored in microfuge tubes containing silica gel as preservative for detection of sporozoites in the salivary glands. The ovaries, blood smear from the mid gut and head and thorax of individual vectors were coded identically for correlating the results. Gravid and unfed *An. fluviatilis* were stored individually in 1.5 ml eppendorf tubes filled with isopropanol after separating the head and thorax for identification of sibling species using allele specific PCR assay and incrimination studies.

### Identification of sibling species

Preserved ovaries of vectors were processed in 50% propionic acid and stained with 2% lacto-aceto orcein according to the method of Green and Hunt [21], for making polytene chromosome preparations. The chromosome complement of individual mosquitoes was examined under a Zeiss Axioplan universal microscope for species-diagnostic inversions used for identification of the members of Fluviatilis Complex [16] and Culicifacies Complex [6]. Body parts of unfed and gravid *An. fluviatilis* stored individually were used for isolation of genomic DNA [22]. The DNA samples were subjected to allele specific PCR assay using species specific primers developed from variable D3 domain of 28S rDNA for differentiation of *An. fluviatilis* sibling species [23].

### Mosquito blood meal source identification

Mid-gut blood smears of *An. fluviatilis* and *An. culicifacies* specimens that were identified to sibling species were subjected to blood meal source identification using human and bovine anti-sera with counter current immunoelectrophoresis [24] and human blood index (HBI) was calculated for each of the sibling species.

### Vector incrimination

Head and thorax of individual *An. fluviatilis* and *An. culicifacies* specimens identified at sibling species level by cytotaxonomic technique/PCR assay were processed for sporozoite detection in the salivary glands. Homogenates of head and thorax of individual mosquitoes in grinding buffer was tested for the presence of circumsporozoite proteins using *P. vivax* (210 & 247) and *P. falciparum* specific monoclonal antibodies by ELISA [25,26].

## Results

A total of 546 female anophelines comprising 8 species were collected during the study period. *An. fluviatilis* was the predominant species accounting for 77.7% followed by *An. jeyporiensis* (11.0%). Man hour density (MHD) of *An. fluviatilis* ranged from 2 to 14 in human dwellings. *Anopheles culicifacies* was encountered in very low numbers with MHD ranging from 0.5 to 3. Other anopheline species prevalent in study villages were *An. aconitus*, *An. sergenti*, *An. splendidus, An. subpictus* and *An. varuna.*

The sibling species composition of the Fluviatilis Complex in the study area as revealed by cytotaxonomy and PCR assay is given in Table 1. Because results were similar in all collections, data were pooled for all the villages surveyed. *Anopheles fluviatilis* species S and T were prevalent in study villages and species U and V were absent. Species S was predominant in the study area comprising 72.9% of the total *An. fluviatilis* samples identified to sibling species. The relative proportion of *An. fluviatilis* species T was less and it accounted for 27.1% of Fluviatilis population screened.

**Table 1:** ***Anopheles fluviatilis *sibling species composition in forest area of Bastar district (Chhattisgarh state)**

**Sibling Species**	**Total Identified**	**Cyto-taxonomy**	AS PCR Assay*	**Relative Proportion**
Species S	306	43	263	72.86%
Species T	114	33	81	27.14%
Species U & V^+^	-	-	-	-

*Allele specific polymerase chain reaction assay.

+ Species U and V were not encountered in study area.

**Figure 2:**
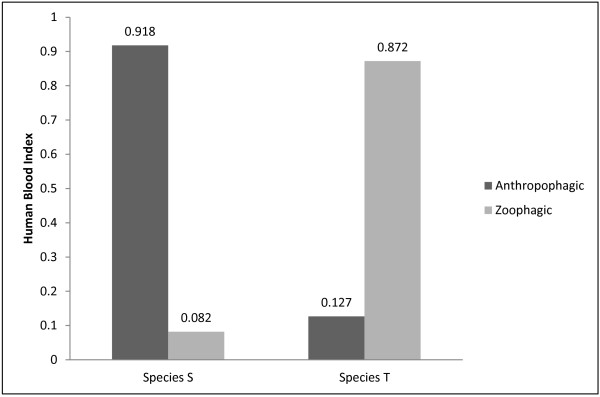
**Feeding preference of *An. fluviatilis *sibling species in forest area of Bastar district (Chhattisgarh state).**

*Anopheles fluviatilis* specimens identified to sibling species were subjected to blood meal source identification using human and bovine anti-sera and the results are depicted in Figure 2. Species S of Fluviatilis Complex was found to be highly anthropophagic as >90% of the samples tested were found fed on humans with HBI of 0.918. In contrast, species T was found to be primarily zoophagic as 87.2% of the specimens were found to have fed on bovine blood, feeding occasionally on humans. The proportion feeding on humans was low and the human blood index was 0.127.

The results of detection of circumsporozoite antigens of *P. falciparum* and *P. vivax* in sibling species S and T by ELISA are given in Table 2. Out of 277 samples of Fluviatilis S tested against *P. falciparum* and *P. vivax* monoclonal antibodies, 6 were found harboring CS antigen of *P. falciparum* with a sporozoite rate of 2.3%. Whereas, none of the specimens belonging to Fluviatilis T was found with circumsporozoite antigen of any of the *Plasmodium* species.

**Table 2:** Detection of circumsporozoite (CS) antigens* of *Plasmodium *species in the members of Fluviatilis Complex by ELISA ^**+**^

**Sibling Species**	**Total tested**	**Total positive**	***Pf***	***Pv*-210**	***Pv*-247**	**Sporozoite rate**
Species S	277	6	6	0	0	2.31
Species T	83	0	0	0	0	0.00

*Circumsporozoite proteins of *Plasmodium falciparum* (*Pf*) and *Plasmodium vivax* (*Pv*-210 & 247).

^+^ Enzyme-Linked Immunosorbent Assay.

Regarding *An. culicifacies* in the study area, as the densities were very low only 28 specimens were identified to sibling species, which revealed prevalence of species B (n=13) and C (n=15) in almost equal proportion. Both the species were found to be exclusively zoophagic as revealed by blood meal source identification and none of the specimens of above mentioned species was found with CS antigen of *P. falciparum* or *P. vivax*.

## Discussion

The state of Chhattisgarh accounts for 2.1% of the total population of the country but contributed 23% of *P. falciparum* cases and 7% of deaths due to malaria in India [27]. About 44% of the geographical area is forested and inhabited by ethnic tribes. These forested areas are the hot bed for intense malaria transmission. Studies carried out in different parts of the state reported *An. culicifacies* and *An. fluviatilis* as major vectors of malaria [5], which have been established as species complexes [6]. Entomological investigations have not been done so far in Chhattisgarh state where these vector species are studied at sibling species level, which is prudent as members of the Fluviatilis and Culicifacies Complexes are known to vary in their biological traits and malaria transmission potential.

Present investigations revealed the prevalence and predominance of *An. fluviatilis* in villages located in the foot hill forest area of district Bastar, Chhattisgarh state. This is because slow running streams and stream channels around the villages are the preferred breeding sites of *An. fluviatilis*[17]*.* Analysis of sibling species composition of *An. fluviatilis* showed that species S and T were sympatric, the former being predominant. This is the first report on the sibling species composition of *An. fluviatilis* from this area. *Anopheles fluviatilis sensu lato* in Bastar area has been reported to be more exophilic than endophilic with low man hour densities in outdoor and indoor collections and in a total sample of 217 specimens no natural infection was reported in this species [4]. The present investigation has been carried out after a gap of about 30 years during which considerable ecological changes have taken place in this area viz., deforestation, increased cultivable area and irrigation. The combined effect of all these factors might have resulted in the change in resting preference of *An. fluviatilis* from outdoors to indoors. In the present investigation, *An. fluviatilis* was found resting indoors in human dwellings. Studies carried out in the neighboring state of Odisha (formerly Orissa) have also shown preference of species S to rest indoors in human dwellings [12,13] and species T has been found resting mainly in cattle sheds in northern India [12,14]. But in the present study, both species S and T were found resting in human dwellings as the cattle sheds were open type without side walls. Such a situation provided equal opportunity to both the species to come in contact with humans but despite this, species S was found to be highly anthropophagic and species T was primarily zoophagic. Distinct differences in the feeding preference can be attributed to innate behavior of the two species and their genetic makeup. In addition to highly anthropophagic behavior, species S specimens were also found positive for *P. falciparum* sporozoites, which further confirms the vectorial efficiency of this species. These observations strongly suggest that Fluviatilis S is playing a major role in malaria transmission in certain forest areas of Chhattisgarh, whereas *An. culicifacies* C, an established vector of malaria [11], might be playing a supportive role when prevalent in good numbers. Species T of Fluviatilis Complex though primarily zoophagic could also contribute to malaria transmission in certain ecological and climatic conditions as this species has been shown to support the sporogonic development of *Plasmodium* species in laboratory experiments [28].

Although *An. culicifacies* has been implicated as principal vector of malaria in central India [29], there are certain foci in hilly and foot hill forest areas where *An. fluviatilis* species S is prevalent and predominant. In such areas Fluviatilis S should be the target species for vector control operation. Mapping the distribution of *An. fluviatilis* and *An. culicifacies* sibling species should be done in malaria endemic districts of Chhattisgarh state and the forest areas that are primarily under the influence of Fluviatilis S need to be delineated. In such areas vector control strategies based on biology and behavior of species S can be formulated that would reduce the densities of principal vector substantially and consequently bring down the malaria incidence.

Regarding vector control options, indoor residual spraying (IRS) with appropriate insecticide with good coverage of human dwellings and encompassing *An. fluviatilis* prevalence period can result in marked reduction in vector population and malaria incidence as reported in *An. fluviatilis* dominated areas of Odisha state [30]. Alternately the use of insecticide-treated nets (ITN), which have been extensively evaluated in many malaria endemic countries against different vector species [31] and the long-lasting insecticidal nets (LN) developed during recent years and evaluated in the field [32-34], would be an effective tool in controlling vectors and malaria. Therefore, in remote inaccessible forest areas where the principal vector is anthropophagic with a preference to rest in human dwellings, public distribution and promotion of use of ITN/LN would certainly reduce vector density and morbidity/ mortality due to malaria.

## Conclusions

The study revealed predominance of species S of the Fluviatilis Complex in forest areas of district Bastar of Chhattisgarh state in central India. Species S was found to be highly anthropophagic and incriminated as a malaria vector. Therefore, remote inaccessible forest areas that are primarily under the influence of Fluviatilis S need to be delineated and this species should be targeted for vector control operation. In such tribal dominated areas, vector control strategies based on biological characteristics of Fluviatilis S would reduce the densities of principal vector substantially and bring down the malaria incidence.

## Competing interests

The authors declare that they have no competing interests.

## Authors’ contributions

NN, RMB conceptualized the study and planned its execution. The manuscript was drafted by NN, with contributions from RMB, SNS and TA. The field work was carried out by SNS and laboratory analysis of the samples for various entomological parameters was done by NN, NPK, PKR, AS and TA. All authors read and approved the final version of the manuscript.
